# A Study of the Particle-Level Fabric and Morphology of Granular Soils under One-Dimensional Compression Using Insitu X-ray CT Imaging

**DOI:** 10.3390/ma11060919

**Published:** 2018-05-29

**Authors:** Md Ferdous Alam, Asadul Haque, Pathegama Gamage Ranjith

**Affiliations:** Department of Civil Engineering, Monash University, Melbourne, Victoria 3800, Australia; ferdous.alam@monash.edu (M.F.A.); ranjith.pg@monash.edu (P.G.R.)

**Keywords:** granular soils, one-dimensional compression, morphology, fabric, X-ray CT imaging

## Abstract

The particle morphology and fabric of a granular soil influence its mechanical behavior. This study focuses on the evolution of the particle-level fabric and morphology of a uniformly graded sand sample subjected to one-dimensional compression up to 64 MPa. The microstructural changes with increased stresses were captured using in situ high-resolution X-ray computed tomography (X-ray CT) imaging. The processed images of particles were separated using the Monash Particle Separation Method (MPSM) for subsequent fabric and morphological analyses. The variations of various fabric parameters were studied using the separated particle volumes. New methods of assessing the morphology and crushability of particles were introduced including a comprehensive algorithm for determining coordination number, branch and contact normal vectors. Results of all fabric parameters were analyzed and discussed with reference to observed changes. Potential mechanisms were identified and relevant correlations were developed where warranted.

## 1. Introduction

The macroscopic behavior of a granular soil subjected to loading is considered to be the reflection of its microscopic response, which is predominantly controlled by soil fabric [1,2]. Soil fabric, which consists of the spatial arrangement of pores, particles and particle groups, experiences continual changes due to load-induced deformation [3,4,5]. The fabric of soil can be described collectively by scalar (void and particle size, coordination number, branch vector length) and vector (particle and void orientation, branch and contact normal vectors) parameters. The influence of soil fabric on the strength and deformation properties of soils have been investigated [5,6,7,8,9,10].

The orientation of the contact normals parallel to the maximum principal stress in the strain hardening stage and a deviated orientation inside the shear zone were reported. Fabric anisotropy derived from the contact normals was found to increase up to the peak stress and then to decrease to a nearly constant value [9]. On the other hand, the mean value of the coordination number of particles was found to be related to the mean value of the void ratio of the assembly [11,12,13]. The standard deviation of the coordination number was linked to the heterogeneity of the fabric, where Hasan & Alshibli [12] reported lesser values of coordination number inside the shear zone compared to the values observed outside the shear zone. Moreover, the void ratios within and outside the shear zones were found to be considerably different from the final void ratio calculated using the global measurements [12,14,15]. The morphology of the particles (sphericity, roundness, surface roughness) is another important factor, which influences the soil fabric [1,16,17,18]. Therefore, an in-depth investigation into the morphology and fabric of soils is of utmost concern in pursuit of linking them to laboratory measured strength and deformation properties.

High stress conditions (c. 350 MPa) can be encountered in many geotechnical applications (deep well shaft, heavy earth dam, blast loading, deep driven pile), which may cause supporting granular soils to crush and develop new fabric and morphology [19,20,21,22]. Particle crushing, consisting of surface grinding, asperity breakage and particle splitting, is associated with applied stress levels [21,23,24,25,26]. A range of methods including experimental, analytical and numerical have been implemented to examine the response of sands under such high stresses [20,21,23,24,25,26,27,28,29,30,31]. However, the integration of modern high-resolution imaging modalities (X-ray, neutron and synchrotron tomography) to conventional geotechnical laboratory experiments is still challenging owing to the large sample sizes and inherent complexities associated with quantitative image analysis (i.e., limitations of segmentation and separation methods, large data handling time, limited fabric analyses tools). Some other techniques (e.g., shear wave velocity measurement) can also be used to assess the fabric anisotropy of soils [32,33]. 

The best possible separation outcome of granular particles subjected to external mechanical stresses is essential for an accurate morphological and fabric analysis [17]. The routinely used marker-controlled watershed separation method has been found to be inadequate in separating particles compressed one-dimensionally under high stresses [34]. The authors introduced the Monash Particle Separation Method (MPSM) based on a cluster analysis-marker-controlled watershed framework to overcome the particle separation issues involved with high stresses and crushing of particles [34]. It is envisaged that the MPSM would enable a positive outcome for morphological and fabric analyses of granular soils, which is one of the prime objectives of this paper. 

Moreover, some recent articles reported results of fabric analysis by discarding smaller-sized particles (50–60 μm), which may evolve from the crushing of particles under high stresses [25,35] and others by accepting the errors [36]. This class of analysis may have a significant effect on both the scalar and vector parameters representing the granular fabric. Therefore, an improved method for analyzing the fabric of granular soils containing particles of smaller sizes is presented. 

This paper investigates the evolution of fabric of sand particles subjected to insitu one-dimensional compression loading up to 64 MPa using X-ray computed tomography (X-ray CT). This load range would allow an in-depth study of granular soil fabric and morphology, where a significant challenge with the separation of particles including generation of fine particles from crushing is highly anticipated. Results of various particle-level fabric parameters were analyzed and appropriate correlations were sought where warranted.

## 2. Materials and In Situ Imaging

Bulk sand samples (medium to very dense, sub-rounded to rounded particles of fine to coarse sizes with uniform to poor gradations) were collected from 13 m depth of the basement excavation for the new Learning and Teaching Building, Monash University, Clayton Campus, Australia. Locally the sands are known as Red Bluff Sands (RBS) of the Brighton group consisting of quartz minerals with trace amounts of iron oxides [37,38]. The collected sand sample was initially wet-sieved using a 75 µm sieve [39] and the retained mass was oven dried for 24 h prior to sieving with 300 and 150 µm sieves [39]. In this study, the sand particles finer than 300 µm sieve and coarser than 150 µm sieve were selected. The average specific gravity of the quartz sand particles measured using a Multipycnometer [40] was 2.65.

**Figure 1:**
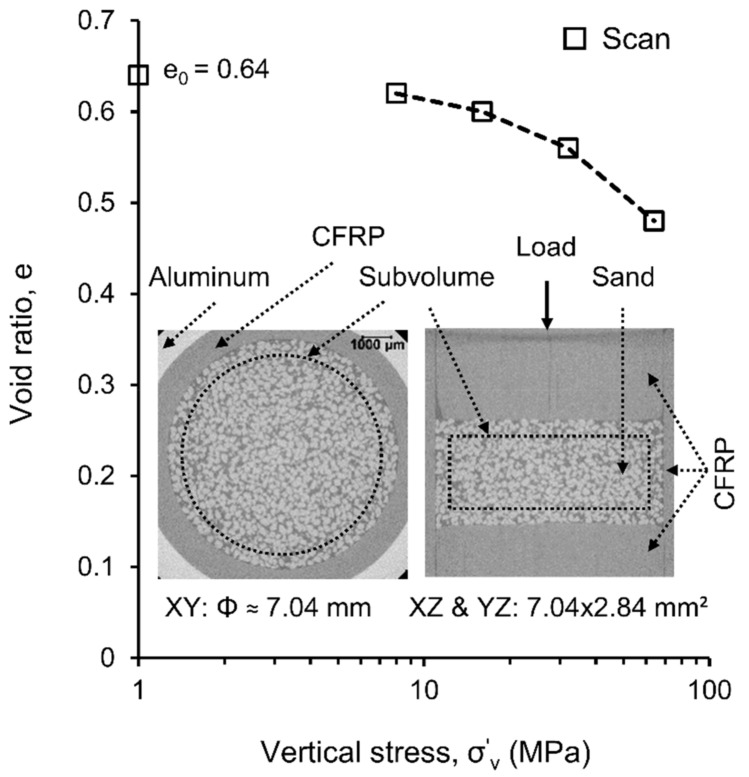
e-log σ’_v_ plot for the specimen consisting of RBS particles tested under one-dimensional compression (Φ: diameter; e_0_: initial void ratio; CFRP: carbon fiber reinforced polymer).

A dry mass of sands of 0.30 g was poured from a height of about 15 mm using a small funnel into a newly designed double-walled (carbon fiber reinforced polymer (CFRP) inner wall and aluminum outer wall) compression cell of 8 mm of internal diameter. A solid rod and base made from CFRP were used as the load plunger and base support respectively. More details on the cell can be found elsewhere [34]. A mild vibration was applied to the cell to achieve a final height of 3.75 mm, which corresponded to an initial dry density of 1613.60 kg/m^3^ and void ratio of 0.64. The whole set up was then placed onto a CT5000 load-stage of 5 kN capacity [41], controlled by MICROTEST (V6.13) software [42] for insitu imaging using the Zeiss Xradia 520 Versa (Xradia, Pleasanton, CA, USA). The compressive load [43] was applied by the upward movement of the bottom platen of the load-stage at a rate of 0.1 mm/min. When the stress reached the desired level, the load was paused and the setup was left for about 30 min to stabilize prior to the imaging. The stress levels selected for this investigation were 0, 8, 16, 32 and 64 MPa (Figure 1). These stresses were selected to capture enough data points near the vicinity of the particle crushing stress [23,44]. The scanning was performed using source-energy and -power of 140 keV and 10 W respectively at 12 µm pixel size by rotating the load-stage 360° around its vertical axis. A total of 1601 projections were taken from which the required three-dimensional greyscale volume was automatically reconstructed using the XRM Reconstructor [45].

## 3. Post-Processing of Images

All images were post-processed using a commercially available image processing software, Avizo [46]. A cylindrical subvolume of 7.04 mm diameter and 2.84 mm height was selected for image analysis (Figure 1). A non-local means filter [46,47] was applied prior to image segmentation. The filtered image volume was then subjected to interactive thresholding by using the intensity threshold value obtained from averaging the threshold values of entropic [46,48,49], factorization [46,50], moment-preserving [46,51] and IsoData [46] methods. The segmented binary image data of the sand volumes were used for further analyses.

## 4. Particle-Level Fabric Analysis

### 4.1. Separation of Particles 

**Figure 2:**
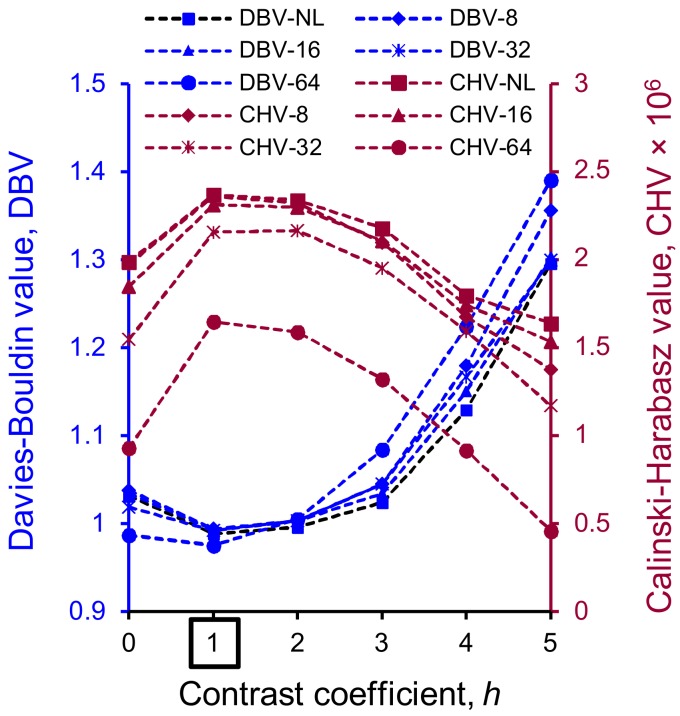
Variation of cluster evaluation criteria values with contrast coefficients (*h*-values) at different vertical stresses (square marked *h*-value represents the optimal condition; NL: No-load).

In order to achieve the best possible separation outcome of the sand particles subjected to loading, the binary volumes were separated using the two-stage Monash Particle Separation Method (MPSM) [34]. Stage one determined the optimal contrast coefficient (h) to be used as an input in the routine marker-controlled watershed method [52,53] based on Calinski-Harabasz [54,55] and Davies-Bouldin [55,56] cluster evaluation criteria. The optimal contrast coefficient [34] for all image volumes was found to be unity (Figure 2). Stage two of the MPSM was implemented to particles, which remained unseparated after the stage one. In this stage, the clustering by Gaussian mixture models [55,57] was used to create cuboid markers for each unseparated particles, which were subsequently integrated to the watershed method for separating particles. Single disconnected voxels (maximum 0.0037% by volume) were removed from the separated volumes for subsequent analyses.

### 4.2. Morphology of Particles

**Figure 3:**
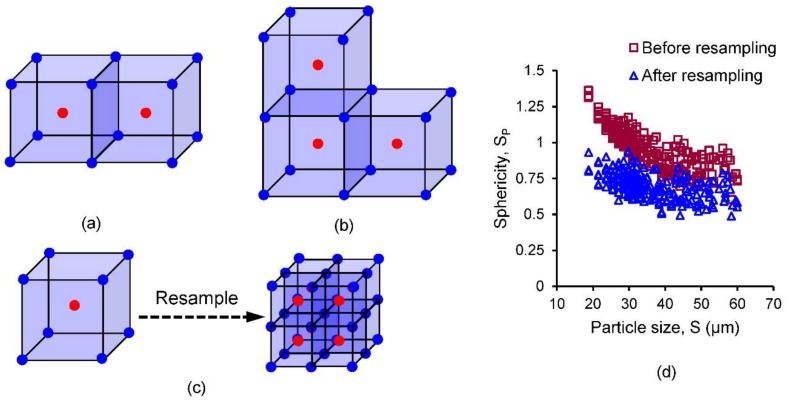
Reasons of erroneous morphological parameters and the proposed solutions: (**a**) voxel centroids on a single line; (**b**) voxel centroids on a single plane; (**c**) resampling; (**d**) comparison of sphericity results for 64 MPa (red and blue circular markers represent voxel centroids and voxel corners respectively).

The morphological change of particles was investigated by determining the sphericity (*S*_p_ = volume-equivalent sphere’s surface area/surface area of the particle) [58] and convexity (*C*_x_ = particle volume/volume of the convex hull). Both sphericity and convexity are measures of compactness of a particle, which are sensitive to both form and roundness [59]. Sphericity calculation was performed in Avizo [46], while convexity was determined using MATLAB [55]. The theoretical values of both sphericity and convexity cannot be greater than unity. However, calculations based on the centroids of voxels may result in erroneous values when the voxel centroids lie on a single line (Figure 3a) or a single plane (Figure 3b). This could happen mostly for smaller-sized particles consisting of few voxels, which may evolve as a result of crushing of particles under high stresses. Therefore, avoidance of such small particles from the morphological analysis may not be appropriate. In this study, resampling of the volumes containing particles of diameter less than or equal to 60 µm was performed in Avizo by dividing a voxel into 8 equal voxels (Figure 3c). Sphericity calculation of the resampled image data produced an excellent sphericity result compared to the non-resampled data (Figure 3d). In addition, for convexity calculation, dividing the particle volume calculated from all of its voxels with the convex volume based on voxel centroids is not justified. Therefore, for all particles, convexity was calculated based on corner points of voxels rather than centroids as shown in Figure 3.

### 4.3. Coordination Number, Branch and Contact Normal Vectors

**Figure 4:**
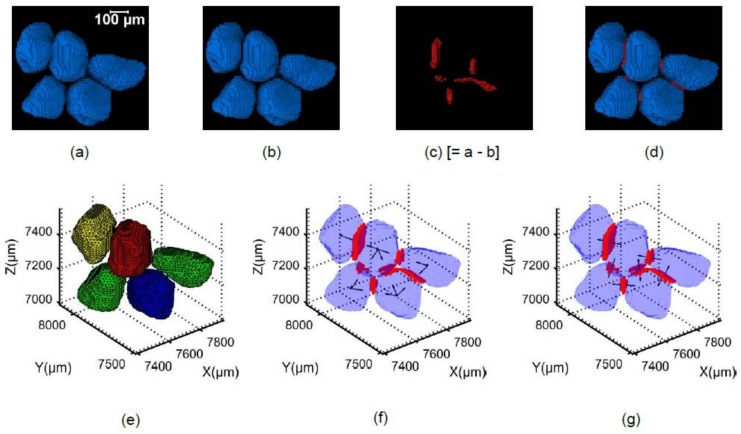
Demonstration of steps involved in determining coordination number, branch vector and contact normal vector: (**a**) unseparated binary particles; (**b**) separated binary particles; (**c**) contact voxels; (**d**) particles with contact voxels; (**e**) coordination numbers (yellow-1, green-2, blue-3, red-4); (**f**) branch vectors; (**g**) contact normal vectors.

The contact voxels between particles were determined by subtracting the binary volume of the separated particles from the binary volume of the unseparated particles (Figure 4a–d). The binary volume containing the contact voxels (Figure 4c) and the labeled volume of the separated particles were exported to MATLAB, where the voxel IDs of the 26 neighborhood of each contact voxel were determined from the separated labeled particle data. Although it is expected that a contact voxel will have only two unique label IDs (except zero) in its 26 neighborhood, very few voxels were found to have more than two IDs, which were discarded. The resultant data was filtered for unique numbers except zero, which was used to identify a contact voxel containing the two unique IDs of the particles that were connected by the contact voxel. The data was then again filtered for unique combinations of 2 particle IDs and searched for the number of particle IDs connected to a specific particle ID, which determined the particle’s coordination number (Figure 4e). The label IDs of the particles, which were in contact with a specific labelled particle were extracted and branch vectors were determined by connecting the centroid of that particle and the centroids of the particles in contact. Subsequently, the branch vectors were converted to unit vectors (Figure 4f) for directional fabric analyses.

**Figure 5:**
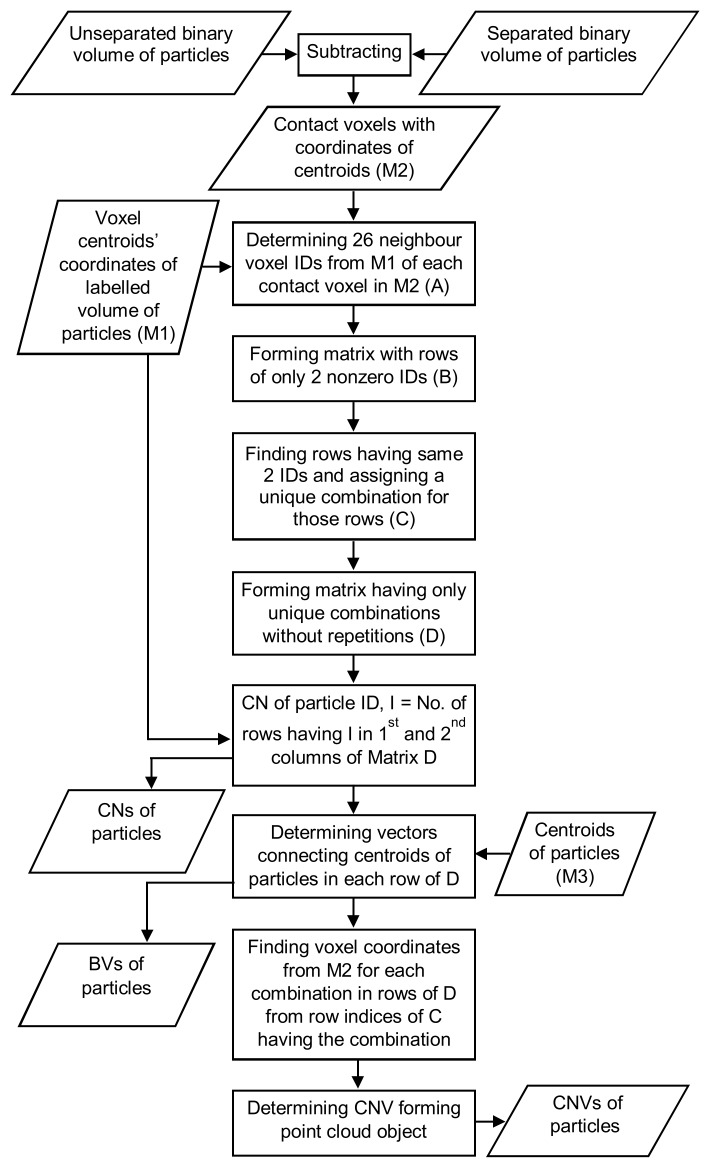
Algorithm flowchart of the code for determining coordination number (CN), branch vector (BV) and contact normal vector (CNV).

The contact voxels having a specific combination of 2 particle IDs were extracted along with their coordinates. A point cloud object was created with those voxel points and their centroid (centroid is the mean of the points) using the intrinsic MATLAB class “pointCloud” [55]. Subsequently, the contact normal vector was calculated at the centroid of those points using the MATLAB built-in function “pcnormals“ by considering all of the points as the neighboring points [55,60]. A contact normal vector could not be found for a single contact voxel [9]. The entire process of developing the code is depicted in Figure 5.

**Figure 6:**
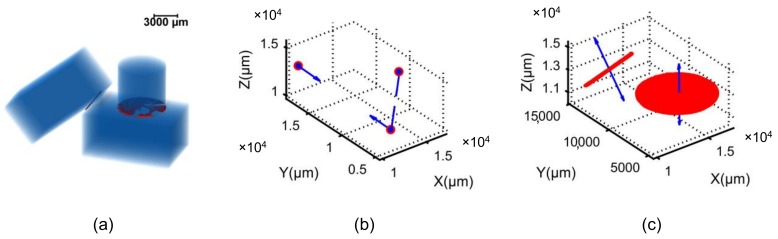
Validation of the code for determining coordination number, branch vector and contact normal vector: (**a**) three regular-shaped aluminum blocks in contact, (**b**) branch vectors, (**c**) contact normal vectors.

The algorithm was initially tested on three regular-shaped aluminum blocks with a simple configuration (Figure 6a–c). As shown in Figure 6a, the contact voxels (red color) between the cylinder and the bottom (flat) cuboid were not properly rendered by Avizo, however, the actual contact surface was rightly shown in Figure 6c as plotted in MATLAB. For the fabric tensor and distribution density calculations, an opposite unit vector was assigned to every contact normal vector as shown in Figure 4g [61].

### 4.4. Fabric Tensors and Distribution Densities

Directional data consisting of particle orientations, branch vectors and contact normal vectors (in terms of unit vectors) were analyzed for fabric tensors and distribution densities. The direction of the major inertia axis (long-axis) of a particle was used to define its orientation which could be extracted as a unit vector from Avizo [46]. In this study, the fabric tensors as described by Kanatani [62] were used. The 4th order approximation of the distribution density (termed as F4-DD) function as defined by Equation (1) was used to represent the intricate distributions of the directional data. In order to find the directional preference of the fabric, an Eigen decomposition was performed on the fabric tensor of the 1st kind of rank 2 as described by Kanatani [62].

(1) $$f\left(n\right)\;=\;\frac{1}{4\pi }F_{ijkl}n_{i}n_{j}n_{k}n_{l}$$

where *F_ijkl_* is the fabric tensor of the 2nd kind of rank 4 [62].

### 4.5. Crushability of Particles from Contact Normal Vectors

**Figure 7:**
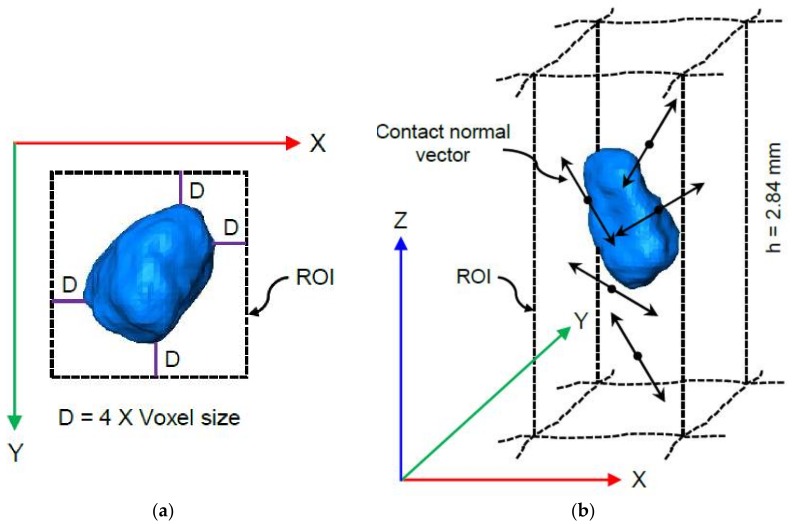
Finding contact normal vectors within a selected region of interest (ROI) around a particle: (**a**) XY view of a particle with the ROI; (**b**) 3D view of a particle with the contact normal vectors in the ROI.

An attempt was made to identify the presence of a strong force chain [26,63,64] from the distribution density of contact normal vectors, which is responsible for particle crushing. A cuboid region of interest (ROI) of 2.84 mm height along the loading direction (i.e., vertical) was formed around a particle by maintaining a fixed distance of 4 times the voxel size from the extreme points of the particle boundary (Figure 7). Contact normal vectors within this ROI were determined (Figure 7b) and the 4th order distribution densities of those vectors were calculated. The maximum value of the distribution density along the loading direction was divided by the minimum value of the two maximum values along the other two axes to define a parameter named as the ‘distribution density ratio (DDR)’. The combined effect of the distribution density and DDR was evaluated to identify the presence of a strong force chain affecting the crushability of a small number of particles. The extension of the ROI to 4 times the voxel size was selected based on the analysis considering the ROI extent up to 10 times, which produced DDR values matching the crushability of particles.

## 5. Results and Discussion

### 5.1. Particle Size Distributions and Morphological Parameters

**Figure 8:**
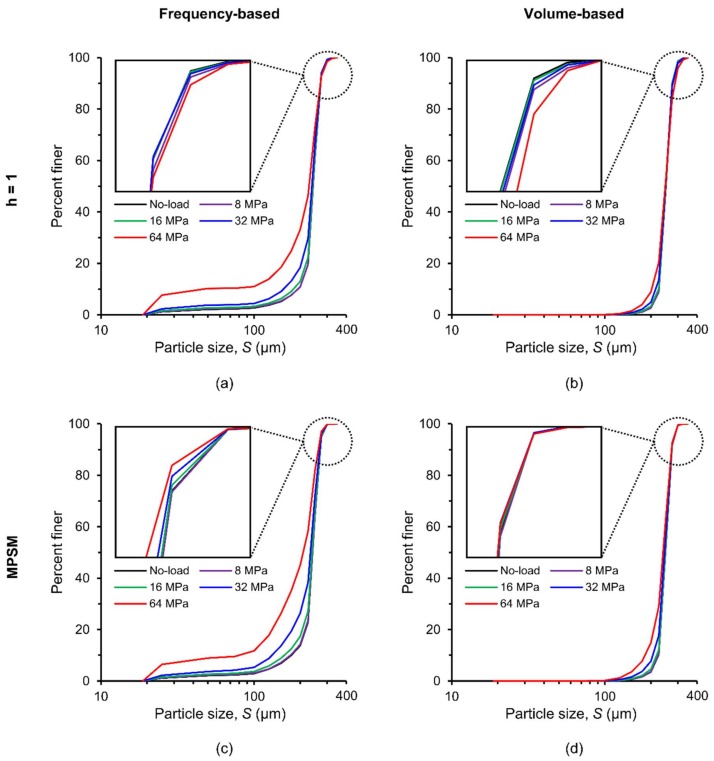
Particle size distributions of RBS particles at different vertical stresses based on: (**a**,**b**) the routine watershed separation method, (**c**,**d**) the Monash Particle Separation Method (MPSM).

Figure 8 shows the evolution of particle size distributions with increasing vertical stresses based on widely used frequencies and volumes of particles using the routine watershed method with optimum contrast coefficient (*h* = 1) (Figure 8a,b) and the MPSM (Figure 8c,d). Theoretically, the particle size distributions of the soil volume containing crushed particles should lie above the initial distribution curve due to the generation of fines unless the maximum possible separation of particles is not achieved. The particle size distributions for large-sized particles (zoom-in views of Figure 8a,b) clearly do not follow right trends, particularly noticeable at higher stresses (64 MPa). The presence of relatively coarser fractions could be a result of inaccurate separation of particles using the routine watershed method. The same particle volumes were separated using the MPSM, which resulted in superior particle size distributions (zoom-in views of Figure 8c,d). Therefore, all subsequent morphological and fabric parameters were calculated from the particle volumes separated by the MPSM only.

As expected, particle size distribution curves after 8 MPa gradually shifted toward the smaller size ranges due to crushing of particles with increasing vertical stresses [21,23,25,30,34,65]. No damage of particles was noticed at 8 MPa stress and a very insignificant damage was noticed at 16 MPa, indicating the crushing stress to be very close to 14 MPa (Figure 1) as reported by Nakata et al. [23] and Al Mahbub & Haque [44]. Significant damage to particles including major splitting occurred under stresses of 32 and 64 MPa, which exceeded the crushing stress. It is also noticeable that particles larger than 240 μm did not experience severe damage even at 64 MPa, which could have resulted from the uniform boundary stresses provided by the smaller particles around them [30].

**Figure 9:**
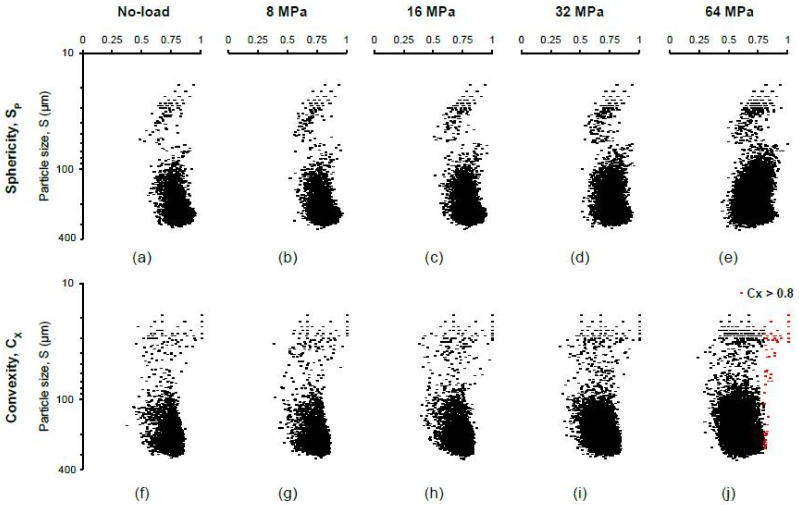
Shape parameters of RBS particles at different vertical stresses: (**a**–**e**) sphericity; (**f**–**j**) convexity.

**Figure 10:**
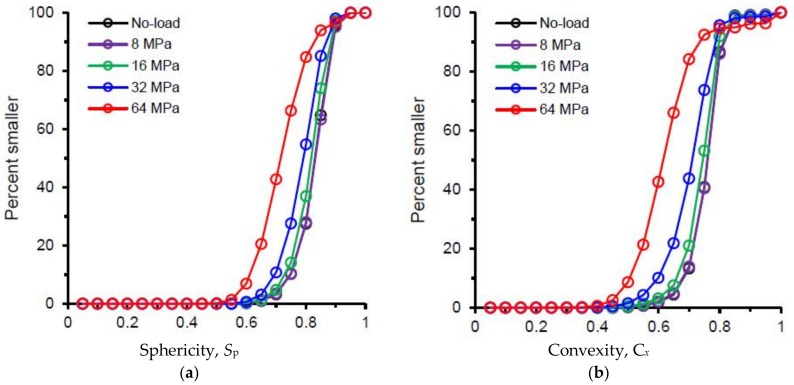
Distributions of shape parameters of RBS particles at different vertical stresses: (**a**) sphericity, (**b**) convexity.

**Figure 11:**
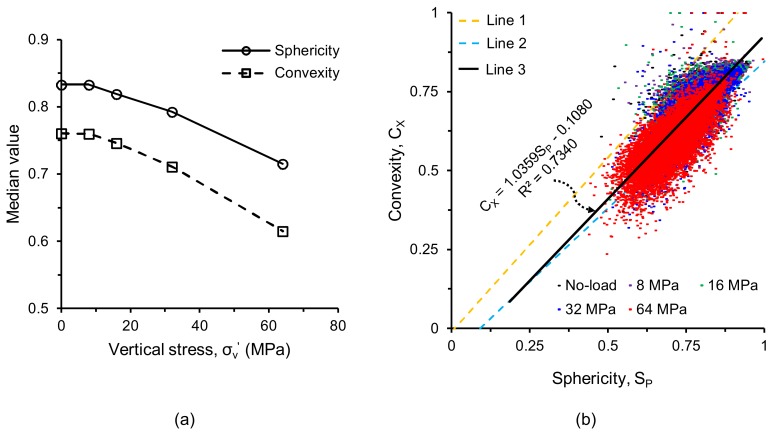
(**a**) Median values of shape parameters of RBS particles at different vertical stresses, (**b**) Correlation between sphericity and convexity of RBS particles (Line 1—Zhao et al. [35], Line 2—Fonseca et al. [36], Line 3—this study).

The two morphological parameters (sphericity and convexity) for all separated particles are plotted in Figure 9. As expected, both the parameters did not show any noticeable variations up to 8 MPa stress (Figure 9a,b,f,g). However, a continuous increase of the size of the black-shaded areas toward the smaller size range of particles after 8 MPa stress was encountered (Figure 9c–e,h–j) due to the progressive crushing of particles with increasing vertical stresses. The newly generated particles after 8 MPa were less spherical and less convex than the original particles as indicated by the leftward shift of their distributions (Figure 10), which is in-line with other outcomes [25,35]. However, at 64 MPa, particles with higher convexity values (*C_x_* > 0.8) were observed to increase (Figure 10b), which could be due to some kind of surface smoothening as reported by Altuhafi & Coop [25] and Al Mahbub & Haque [44]. The analysis also showed that most of the particles with convexity values larger than 0.8 existed in the lower range of particle sizes (Figure 9j). A close inspection of the image volumes revealed that the smaller size particles of few voxels lying on a single line or a single plane resulted in higher convexity values with lower sphericity values. A decreasing trend of median values of the sphericity and convexity parameters with increasing vertical stresses (post-crushing stage) was observed in this study (Figure 11a), which was also reported by Zhao et al. [35]. A plot of the convexity vs. sphericity values of all particles for the stress-range tested (Figure 11b) showed an acceptable correlation (*R*^2^ = 0.734) when compared with other researchers [35,36].

### 5.2. Particle Long-Axis Orientations

**Figure 12:**
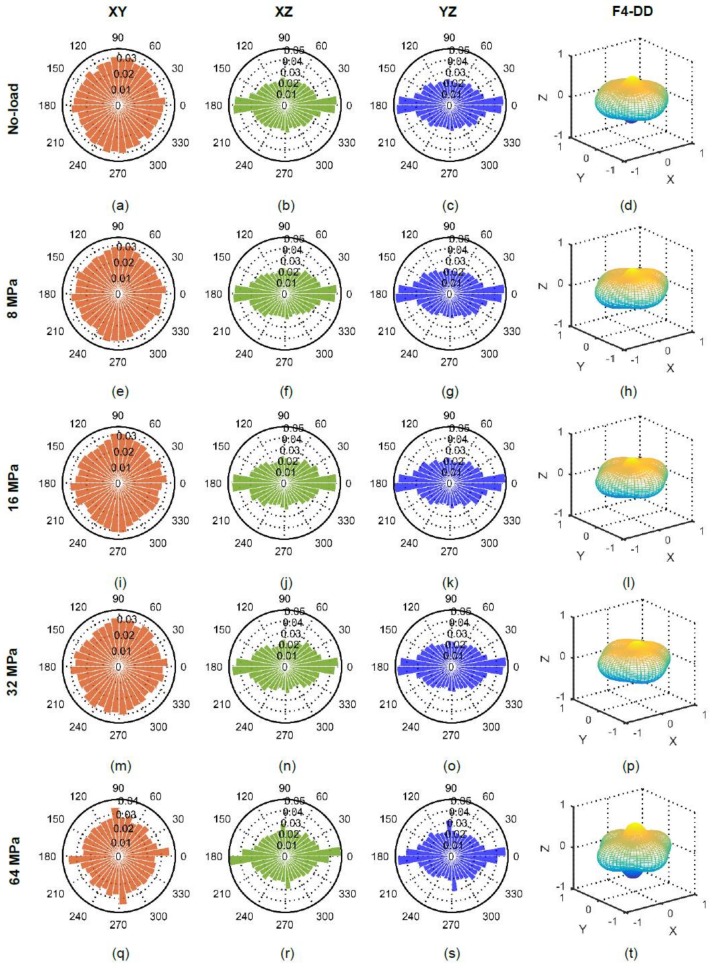
Particle orientations at different vertical stresses for RBS particles (F4-DD: 4th order approximation of distribution density).

**Figure 13:**
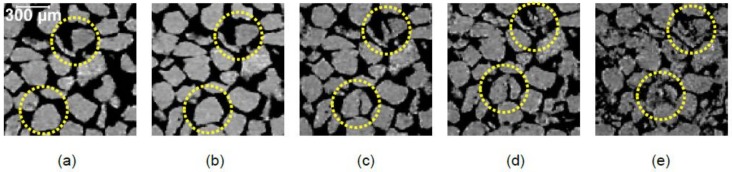
Crushing of RBS particles with increasing vertical stresses: (**a**) no-load; (**b**) 8 MPa; (**c**) 16 MPa; (**d**) 32 MPa; (**e**) 64 MPa.

The orientations of particles’ long-axes in XY, XZ and YZ planes along with their distribution densities for all applied stresses are plotted in Figure 12. The initial (no-load) orientations of particles (Figure 12a–d) showed a greater horizontal bias (perpendicular to the loading direction) resulting from the packing of particles under the action of gravity [8]. Moreover, some particles were found to have their major axes nearly vertical, which could have been occurred from the inclusion of cut-portion of particles in the subvolume analyzed. The dominance of horizontally biased orientations continued up to 32 MPa (Figure 12e–p) thereafter a vertical bias appeared at 64 MPa (Figure 12q–t). The severe crushing of particles at 64 MPa led to particles splitting [26] as evidenced in Figure 13.

### 5.3. Particle Coordination Numbers

**Figure 14:**
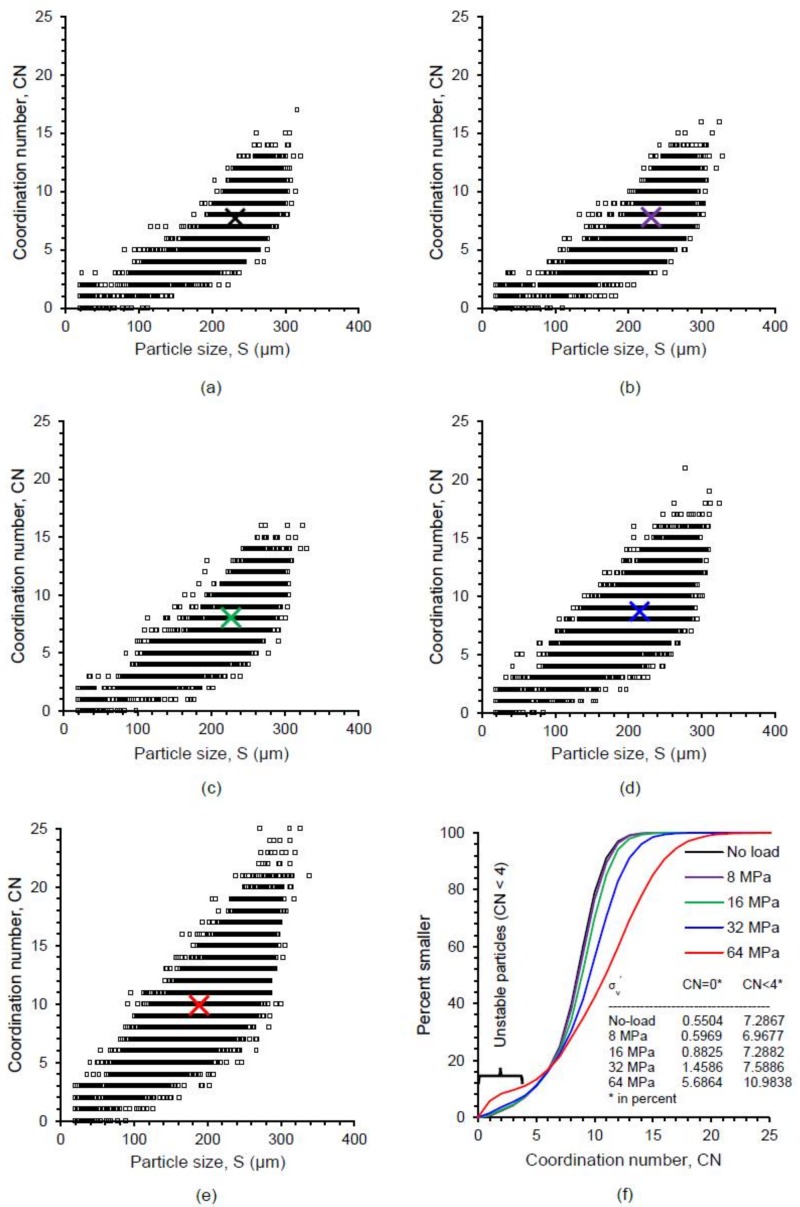
Coordination number of RBS particles at different vertical stresses: (**a**) No-load; (**b**) 8 MPa; (**c**) 16 MPa; (**d**) 32 MPa; (**e**) 64 MPa; (**f**) distributions of coordination number (X marks represent data centroids).

**Figure 15:**
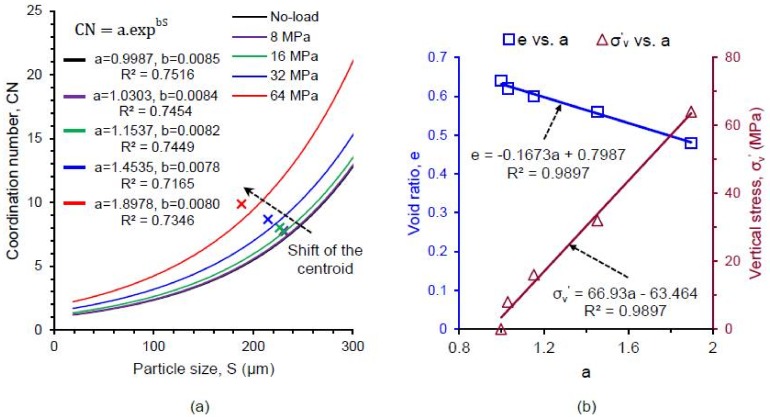
(**a**) Correlation between particle size and coordination number at different vertical stresses for RBS particles; (**b**) Correlation between parameter “*a*”, vertical stress and void ratio of RBS particles.

The coordination number of a particle plays a vital role in its crushing behavior, which has also been confirmed by discrete element method [64,66]. Figure 14 shows the distributions of the coordination number of particles with their sizes at different vertical stresses (no-load, 8, 16, 32 and 64 MPa). In general, the coordination number was found to increase with the increase of particle size and applied stress (Figure 14a–e). An insignificant proportion (0.55–1.46% by number) of smaller particles was found to have a coordination number of zero up to 32 MPa and a relatively high proportion of 5.69% at 64 MPa (Figure 14f), which could be linked to unavoidable image noises, boundary effects, and minor errors in the marker-controlled watershed separation method. The number of unstable particles with coordination number less than 4 [11] remained almost unchanged (≈7%) up to 32 MPa, after which the number of unstable particles increased noticeably (≈11%) due to the severe crushing of particles at 64 MPa. The coordination number distributions with various stresses showed a gradual rightward shift indicating an overall increase of coordination number with the increase of vertical stresses (Figure 14f). The observed exponential variations of coordination number with particle sizes at various stresses could be correlated using an equation in the form of CN = a.exp^b.S^, where a, b are constants, CN is coordination number and S is particle size in µm (Figure 15a). The data centroid locations plotted on Figure 14 and Figure 15a showed a gradual leftward and upward shift indicating the crushing and increase of coordination number of particles due to increasing vertical stresses. Interestingly, the value of the ”*b*“ parameter for the soil and stress-range investigated showed almost a constant value (0.0078–0.0085), which could be related to the narrow variations of median values of morphological parameters of sand particles (Figure 11a). However, the “*a*” parameter showed a noticeable change of values from 0.9987 to 1.8978 with increasing stresses. The variation of “*a*” parameter was found to linearly increase with the increase of vertical stress and decrease with the reduction of the void ratio (Figure 15b).

**Figure 16:**
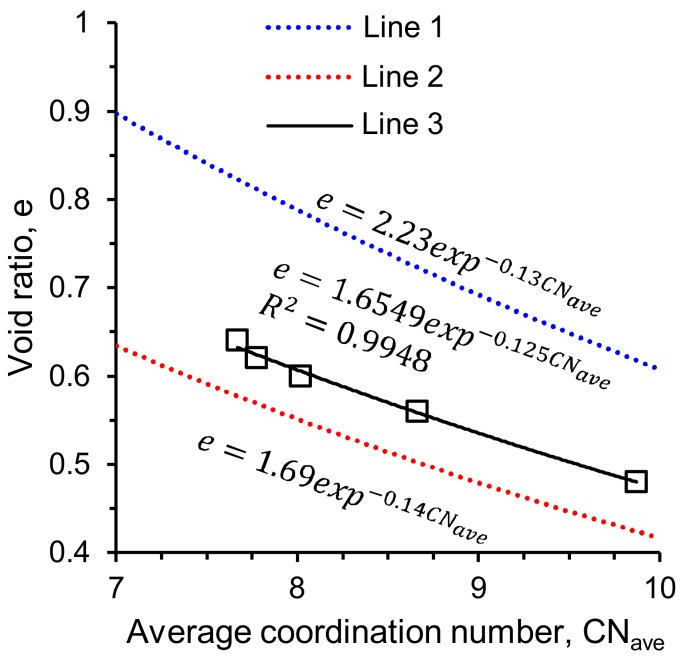
Correlation between average coordination number and void ratio of RBS particles (Line 1—Hasan & Alshibli [12], Line 2—Fonseca et al. [13], Line 3—this study).

**Figure 17:**
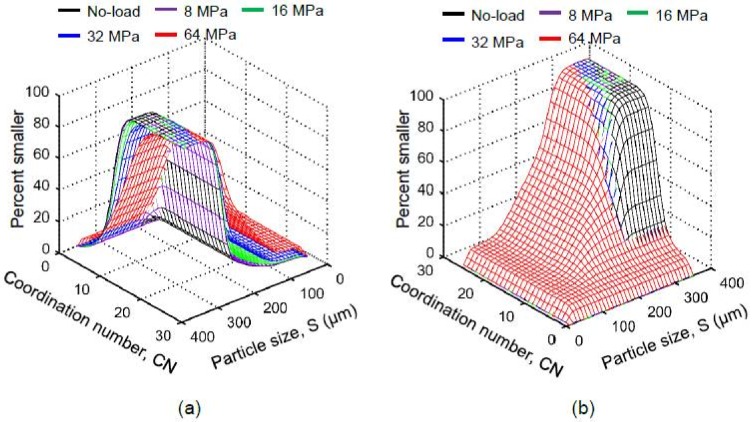
Particle size dependent distributions of coordination number for RBS particles at different vertical stresses: (**a**) view-1; (**b**) view-2 (180° rotation of view-1).

This study also found a good correlation between the void ratio (e) and average coordination number (CN_ave_) in the form of e = c.exp^−d.CNave^ (Figure 16), which fitted reasonably well within the upper and lower bound relationships of Hasan & Alshibli [12] and Fonseca et al. [13] respectively. Figure 17 shows a complete picture of the underlying micromechanics of particle crushing by combining the variations of coordination number with particle size distributions at various stresses. It is interesting to note that the coordination number only increased for particles larger than or equal to 240 μm, below which the distribution curves shifted outward with increasing stresses. This means that particles larger than or equal to 240 μm did not undergo severe damages due to the support provided by many neighboring crushed fragments [30].

**Figure 18:**
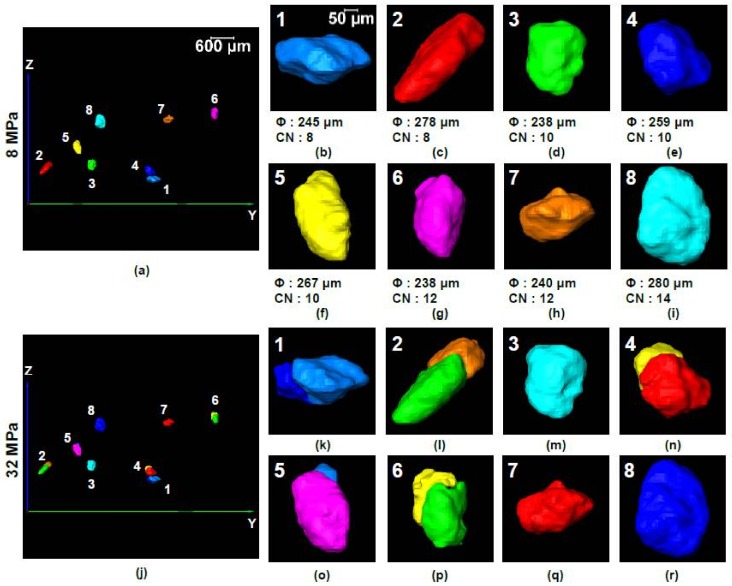
Relation between coordination number and crushing of particle: (**a**) YZ view of selected particles at vertical stress of 8 MPa; (**b**–**i**) zoom-in views with coordination numbers and sizes of particles 1 to 8 respectively at 8 MPa; (**j**) YZ view of corresponding particles at vertical stress of 32 MPa; (**k**–**r**) zoom-in views of particles 1 to 8 respectively at 32 MPa.

The interplay between coordination number and particle size has been further explained by tracking particles from the pre-crushing stress (8 MPa) and post-crushing stress (32 MPa). Eight particles of different sizes and coordination numbers were selected randomly (Figure 18a,j). It was very difficult to track corresponding particles at 64 MPa because of severe crushing. In the selected particle group, particle-3, 4 and 5 (Figure 18a,d–f) had the same coordination number of 10 at 8 MPa, but the smallest of them (particle-3) did not crush at 32 MPa (Figure 18m) because of being stronger [23,24] than the other two, which crushed (Figure 18n,o). Another important factor affecting the crushing of particles is their relative positions with respect to the loading piston position, where a particle close to the piston would have a greater chance of crushing assuming the size and coordination number are the same [26]. Particle-6 and 7 of nearly equal size had the same coordination number of 12 (Figure 18g,h) at 8 MPa, but particle-6 underwent crushing at 32 MPa (Figure 18p) due to its position near to the loading piston. Particle-3 and 6 (Figure 18d,g) were similar in size, but particle-6 crushed due to its position closer to the loading piston although it had higher coordination number (Figure 18m,p). Particle-2 and 8 (Figure 18c,i) had nearly the same size, but particle-2 crushed (Figure 18l) because of its lower coordination number (CN = 8) compared to particle-8 (CN = 14) (Figure 18r). Both particle-1 and 4 underwent crushing (Figure 18k,n) because they were very close to each other and crushing of any one of them instigated the crushing of the other one [26]. Because of this neighboring particle influence, particle-1 crushed (Figure 18k) although it was smaller and had the same coordination number (CN = 8) compared to particle-2 (Figure 18c).

### 5.4. Branch and Contact Normal Vectors

**Figure 19:**
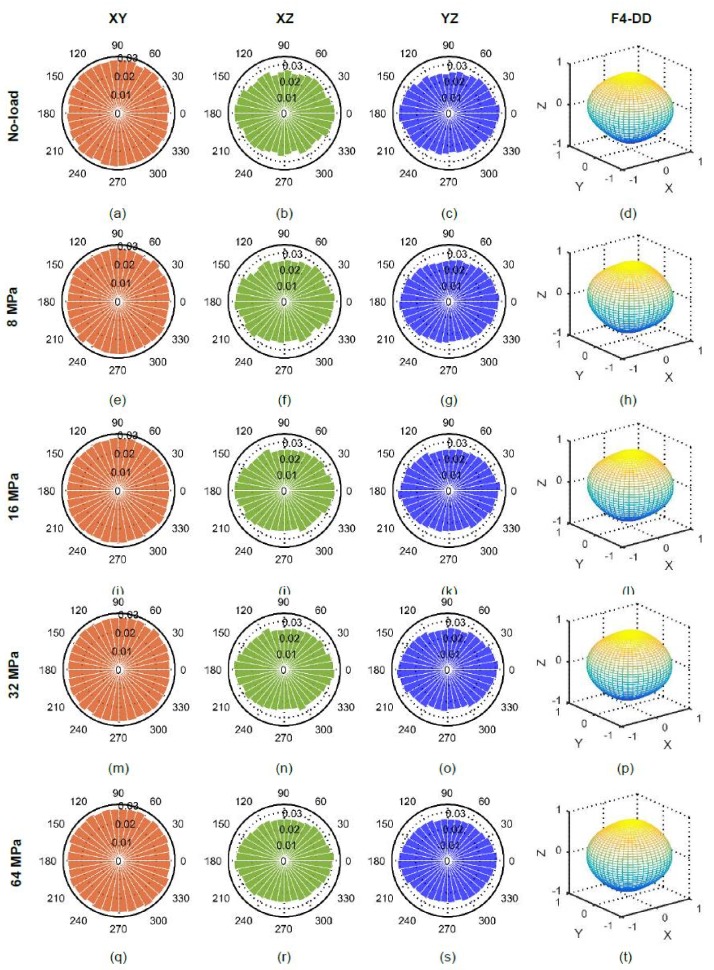
Branch vectors of RBS particles at different vertical stresses (F4-DD: 4th order approximation of distribution density).

**Figure 20:**
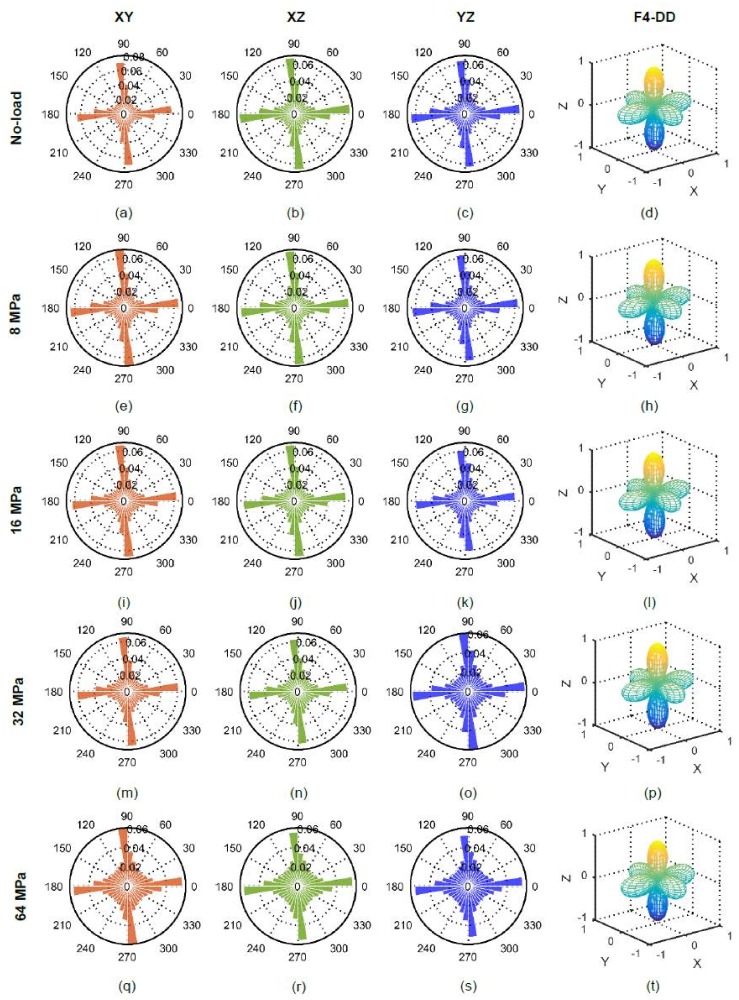
Contact normal vectors of RBS particles at different vertical stresses (F4-DD: 4th order approximation of distribution density).

**Figure 21:**
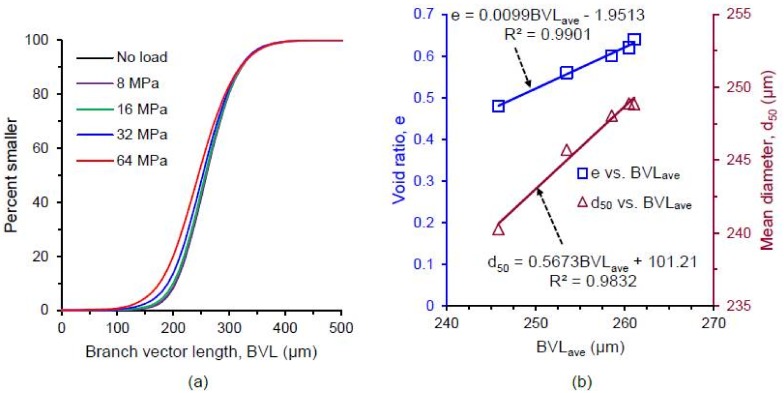
(**a**) Distributions of branch vector length of RBS particles at different vertical stresses; (**b**) Correlation between average branch vector length (BVLave), void ratio and mean diameter of RBS particles.

Figure 19 and Figure 20 show the evolution of branch and contact normal vectors respectively in XY, XZ and YZ planes along with their distribution densities with the increase of vertical stress. It is clear that there was no significant preferred orientation of the fabric of branch vectors (i.e., nearly isotropic). However, the lengths of the branch vectors gradually decreased at post-crushing stresses (≥16 MPa) as shown in Figure 21a due to the crushing of particles, which resulted in a reduction of particle size, also observed by Fonseca et al. [13]. Strong proportional correlations existed between the average branch vector length (BVL_ave_) and void ratio (e), BVL_ave_ and mean diameter of particles (d_50_) as shown in Figure 21b.

Unlike branch vectors, contact normal vectors showed great horizontal and vertical biases at all vertical stresses (Figure 20). The vertical bias of contact normal vectors resulting from the formation of load columns in the loading direction is expected as has been reported by others [5,6,7,8,9]. However, the horizontal bias as observed under one-dimensional compression could also be possible due to the lateral confinement. The Eigen decomposition of the 1st kind-rank 2 fabric tensor [62] derived from the contact normal vectors resulted in a preferred direction of 3.73° from the horizontal plane (XY plane) at no-load condition. Interestingly, this angle became 63.45° when the stress increased to 8 MPa indicating the formation of strong vertical load columns in the system. This vertical bias continued up to 32 MPa, after which the angle became 8.14° at 64 MPa indicating again horizontal bias. At 64 MPa, many nearly vertical fractures were observed (Figure 13e), some of which might not fully separate the crushed fragments and some of which were just single or two voxels in width. Therefore, contact voxels might be found when watershed lines passed through those fractures resulting in nearly horizontally biased contact normal vectors. Many contact normal vectors at 32 and 64 MPa were found oriented in the directions between horizontal and vertical resulting in the bulged geometric shapes of distribution density compared to other stresses as evidenced in Figure 20m–t.

### 5.5. Effect of DDR on the Crushing Potential of Particles

**Figure 22:**
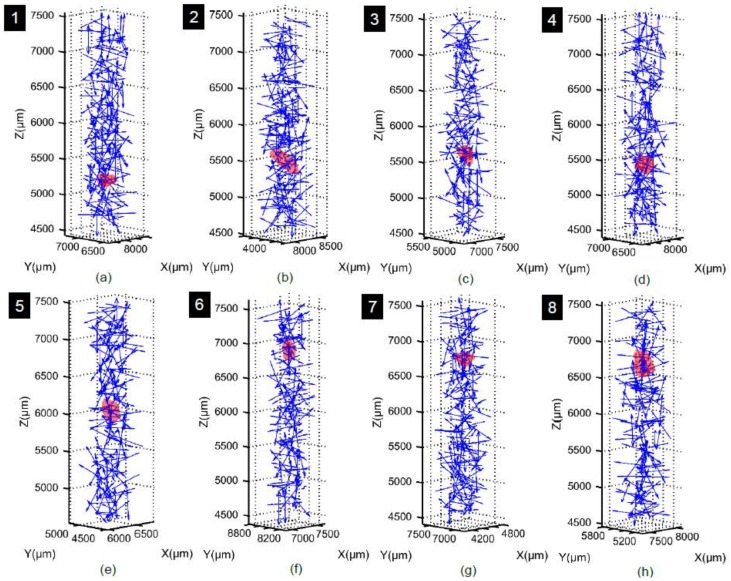
Contact normal vectors within selected regions of interest around particles at vertical stress of 8 MPa (numbers with black background represent the particle IDs of Figure 18; red regions represent the particles’ positions).

**Figure 23:**
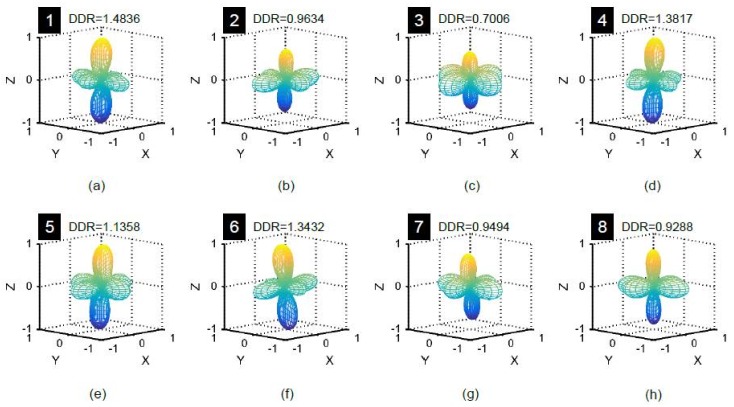
4th order approximation of distribution density for contact normal vectors within selected regions of interest around particles at vertical stress of 8 MPa (numbers with black background represent the particle IDs of Figure 18).

Figure 22 presents the contact normal vectors within the ROI (Figure 7) for the eight particles considered in Figure 18 at 8 MPa. Careful observation of these vectors confirms that most of the vectors were nearly oriented toward the vertical loading direction for particle-1, 2, 4, 5 and 6, which crushed at 32 MPa. There were many horizontally biased contact normal vectors for particle-3, 7 and 8, which did not crush. The relatively dominant vertical bias of the contact normal vectors manifesting strong force chains could be responsible for crushing of particles, a phenomenon also successfully demonstrated with the help of discrete element modeling [26]. For further clarification, the distribution densities (F4-DD) for the contact normal vectors within the ROIs were calculated and are shown in Figure 23. The shapes of the distribution densities of the crushed particles were predominantly vertically biased with distribution density ratios (DDR) greater than unity except that of particle-2. Particle-2 is an elongated particle (Figure 18c), which made it vulnerable to crushing. Based on the limited analyses carried out, it can be hypothesized that DDR could be an important parameter for studying the crushability of particles.

## 6. Conclusions

A one-dimensional compression test on a uniformly graded silica sand sample was conducted up to a vertical stress of 64 MPa and insitu X-ray computed tomography imaging was performed to capture the microstructural changes of soils. The image data was comprehensively analyzed for the particle-level morphology and fabric of granular soils, where the Monash Particle Separation Method (MPSM) played a significant role in separating the particles. The outcomes of the fabric analyses are summarized below:

- The MPSM was capable of separating the granular particles subjected to high stress levels, whereas the routine marker-controlled watershed method failed to produce the maximum separation outcomes. The particle size distributions showed no/insignificant damaged particles in the pre-crushing stress range (<16 MPa) whereas major damage including splitting was noticed in the post-crushing stress range (≥16 MPa).
- The proposed resampling of image data for the smaller-sized particles, whose voxel centroids might lie on a single line or a plane, was found to produce meaningful sphericity values (≤1), which otherwise been ignored for morphological analysis. In general, the morphological parameters (sphericity and convexity) showed significant changes in the post-crushing stress-range due to the creation of smaller-sized particles of smaller sphericity and convexity values. The median values of the convexity and sphericity were observed to decrease with the increase of vertical stresses. This study found a linear relationship between the convexity and sphericity values.
- A comprehensive algorithm for determining the coordination number, branch and contact normal vectors of particles was developed. This study found a dominant horizontally biased orientation of particles’ long-axes up to 32 MPa, thereafter a vertical bias appeared at 64 MPa as a result of many nearly vertical splitting of particles. In general, the coordination number was found to increase with the increase of particle size and applied stress. A good correlation between the void ratio and the average coordination number was observed.
- The micromechanics of particle crushing was described by combining the variations of coordination number with particle size distributions at various stresses, where an increase of coordination number for particles ≥240 µm was observed due to the additional support provided by the newly created particles as a result of crushing. The crushing potential of particles was explained with the help of coordination number, particle size and position relative to loading piston.
- The evolution of branch vectors under one-dimensional compression was found to be nearly isotropic with a gradual decrease of length in the post-crushing stress range (≥16 MPa). A strong correlation was evident between average branch vector length and void ratio, and mean diameter of particles. The contact normal vectors, on the other hand, showed significant horizontal and vertical biases at all stress levels investigated. Analysis of 1st kind-rank 2 fabric tensors showed a significant shift of the orientation of contact normal vectors from the horizontal at no-load to nearly vertical (i.e., formation of load columns) up to 32 MPa, thereafter again horizontal bias at 64 MPa.
- This study reports an innovative way of assessing the crushing potential of a particle by determining the distribution density of contact normal vectors around that particle. In general, prior to crushing, the distribution densities were predominantly vertically biased for crushed particles with distribution density ratios exceeding unity.

The outcomes of this study provide valuable insights of the evolution of particle-level morphology and fabric of granular soils subjected to one-dimensional compression. However, the void fabric, another important component of the granular fabric, requires equal attention. The authors are in the process of developing a new 3D scan-line method for studying the void fabric, which will be addressed in a separate paper.
